# Physical exercise: bulking up neurogenesis in human adults

**DOI:** 10.1186/s13578-019-0337-4

**Published:** 2019-09-03

**Authors:** Xinjuan Lei, Yajun Wu, MengMeng Xu, Odell D. Jones, Jianjie Ma, Xuehong Xu

**Affiliations:** 1 National Engineering Laboratory for Resource Development of Endangered Crude Drugs in Northwest of China/CGDB, Shaanxi Normal University College of Life Sciences, Xi’an, 710119 China; 2 Department of Pediatrics, Columbia University, New York, NY 10032 USA; 3 University of Pennsylvania University Laboratory Animal Resources, Philadelphia, PA 19114 USA; 4 Ohio State University College of Medicine, Columbus, OH 43210 USA

**Keywords:** Neurogenesis, Adult, Physical exercise, Cognitive function

## Abstract

Whether neurogenesis occurs in the adult human brain has been a long-debated topic fueled by conflicting data both for and against neurogenesis in the mature brain. Recent reports from two independent teams may have indubitably proven that adult, hippocampal neurogenesis persists throughout the human lifespan. Llorens-Martín et al. found that thousands of immature, neurogenesis related, doublecortin-positive (DCX^+^) labelled neurons can be detected in the human dentate gyrus (DG) up to the eighth decade of life. While the presence of these DCX^+^ neurons decrease with age, they are significantly decrease in patient with Alzheimer’s disease. Another group have also found mammalian embryonic Hopx^+^ precursors to persist beyond the early development stage as quiescent Hopx^+^ radial glial-like neural progenitors during early postnatal period, then as Hopx^+^ adult dentate neural progenitors. Together, the findings from these two groups suggest that unlike the previously thought, neurogenesis and neuroplasticity can occur well into adulthood in some capacity, at least in the hippocampus. These recent findings that neurogenesis can occur beyond development have brought into questions whether physical exercise can be shown to promote neurogenesis and brain health, as it has been shown to promote the function of other organ systems. Some data has already shown physical exercise to induce adult hippocampal neurogenesis (AHN) as demonstrated by restoration of cognitive functions, improvement of synaptic plasticity, and enhancement of angiogenesis. A large-scale meta-analysis has also demonstrated that 45–60 min of moderate-intensity physical exercise to dramatically improve cognitive functions in human subjects over the age of 50. Given these convergent developments in our understanding of neurogenesis and exercise induced improvement in cognitive function, we speculate that hippocampal neurogenesis can be promoted by physical exercise and discuss the current molecular evidence supporting the likely molecular pathways involved.

Although it has been reported that the recruitment of young neurons to the primate hippocampus decreases sharply after the 1st year of life [1], many contradicting studies have found neurogenesis to persist in some parts of the adult hippocampus. Recent investigations confirmed neurogenesis to exist in adult human subjects [2, 3] while others have demonstrated that physical exercise can significantly strengthen intracerebral retrieval of synaptic plasticity and promote cardiovascular protection via myokine FNDC5/irisin-αV/β5 integrin signaling [4–6]. These findings have encouraged the investigation of physical exercise to strengthen or recover synaptic plasticity by encouraging the differentiation of new neuronal cells.

While neurogenesis has been well-documented in mature invertebrates, fish, reptiles, birds, and non-human mammals [7], a lack of solid evidence in humans has made the existence of neurogenesis in the adult human a controversial topic until recently. This year, using state-of-the-art tissue procedures, Llorens-Martín and colleagues from Spain have identified thousands of doublecortin-positive (DCX^+^) immature neurons in the dentate gyrus of healthy adults between the ages of 43 to 87 and compared them to the significantly fewer DCX^+^ found in Alzheimer’s disease patients [2]. As the only structure in the adult mammalian known to develop new neurons throughout life, the adult hippocampus displays a unique plasticity due to adult hippocampal neurogenesis (AHN). Llorens-Martín et al. has found AHN to be active in the healthy adult up to the ninth decade of life, whereas the number and maturation of these neurons are decreased in Alzheimer’s disease patients and further declines with disease advancement. Similarly, an earlier collaborative investigation between labs at Columbia University and Cyril & Methodius University reported adult hippocampal neurogenesis in healthy human subjects ranging from 14 to 79 years of age [3]. Surprisingly, this group found comparable numbers of intermediate neural progenitors, Ki-67 and nestin labeled immature neurons, glia, mature granule neurons, and dentate gyrus (DG) volume across all ages, with slightly less angiogenesis and a smaller pool of quiescent progenitors in the anterior-mid DG of human aging [3]. Their data claimed that healthy older human individuals preserved their hippocampal neurogenesis throughout life if they are free of cognitive impairment, neuropsychiatric disease, medication and drug use.

While the existence of adult neurogenesis in the hippocampus has proven to be a persistent feature of healthy adulthood and aging, the existence of this process is debated and sometimes contributed to neuroplasticity [8]. However, multiple studies using antibodies against the immature neuron marker protein, DCX, have found potential neuronal progenitors throughout the adult human dentate gyrus [2–4] while other studies report a distinct lack of neuronal generation in the adult hippocampus [1]. These conflicting results between independent groups could be contributed to the incomplete binding of the DCX antibody to the protein found in the human brain [9]. Further data supporting the existence of neurogenesis in the adult hippocampus comes from Berg et al. at Johns Hopkins University, who reported that Hopx+ precursors found at embryonic day 11.5 to be the embryonic origin of adult dentate neurogenesis [9]. These Hopx+ precursor cells in the mouse dentate neuroepithelium develop to become proliferative Hopx+ neural progenitors, then transition to Hopx+ quiescent radial glial-like neural progenitors (QNPs) during the early stages of postnatal development [9]. These QNPs are a new fundamental basis of adult dentate neurogenesis likely preserved in all mammals, including humans.

Co-regulation of AHN and angiogenesis is well-recognized as a critical issue on DG function and volume [3]. Using MRI based measurements of cerebral blood volume (CBV) in mice after 2-weeks of voluntary running in 2007, Pereira and colleagues documented significant exercise-induced increases in dentate gyrus CBV that correlated with postmortem measurements of increased neurogenesis. This exercise-induced CBV boost also correlated with enhanced cognitive function and the expected cardiopulmonary improvement. This finding was corroborated in eleven human subjects ranging from 21 to 45 years old, where increased CBV in the hippocampal dentate gyrus indicating neurogenesis was found after 1 h of aerobic training. These results in combination with exercise-induced retrieval of intracerebral synaptic plasticity via myokine FNDC5/irisin-αV/β5 integrin signaling in human subjects, strongly supports the possibility of physical activity promoting neuronal regeneration in the adult brain [4–6]. Meta-analysis from 12,820 records of adults over the age of 50 further support this conclusion by demonstrating that cognitive function can be improved by regular sessions of 45–60 min of moderate intensity physical exercise [10]. These mechanistic, physiologic, and health statistics findings all demonstrate that just as physical exercise can improve cardiovascular health, it may also improve cognitive capacity by promoting protective and restorative neurogenesis (Fig. 1).

**Fig. 1:**
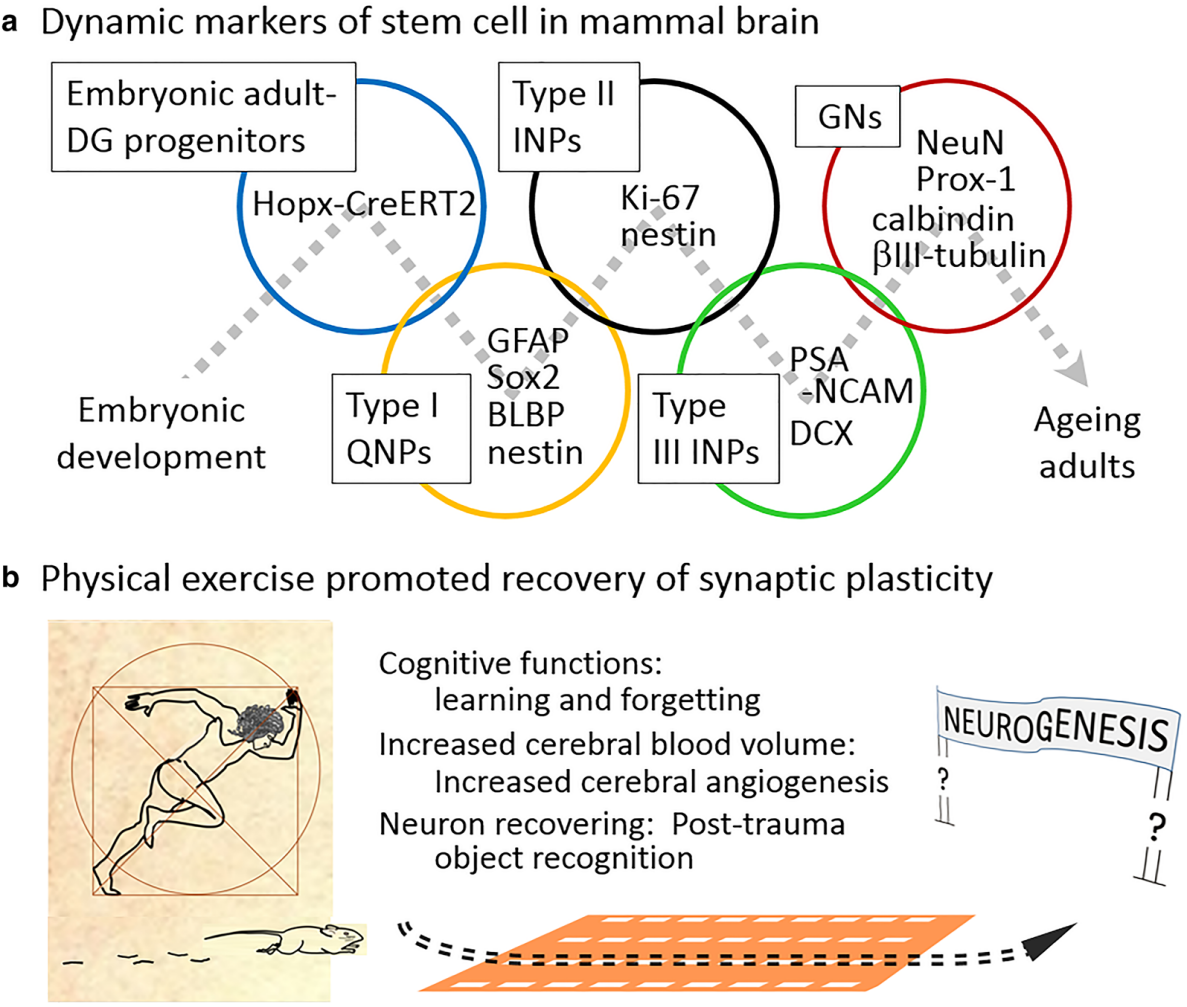
Known pathways in neurogenesis and exercise promoted neurological benefits. **a** Embryonic origin of mature granule neurons (GNs) originate from adult-dentate gyrus (DG) progenitors derived from common embryonic stem cells. DGs differentiate into quiescent radial-glia like type I neural progenitor cells (Type I QNPs), then type II intermediate neural progenitors (Type II INPs), then Type III INPs, which eventually differentiate into immature and mature granule neurons (GNs). The genes and proteins used to label each differentiation stage are shown in the circle of each progenitor stage; **b** known physical exercise-promoted enhancement and recovery of cognitive functions

## Data Availability

Not applicable.

## Abbreviations

AHN: adult hippocampal neurogenesis
CBV: cerebral blood volume
DCX^+^: doublecortin-positive
DG: dentate gyrus
FNDC5: fibronectin type III domain-containing protein 5
GNs: granule neurons
QNPs: quiescent radial glial-like neural progenitors
Type I QNPs: quiescent radial-glia like type I neural progenitor cells
Type II INPs: type II intermediate neural progenitors
Type III INPs: type III intermediate neural progenitors
